# Naringenin, a citrus flavanone, enhances browning and brown adipogenesis: Role of peroxisome proliferator-activated receptor gamma

**DOI:** 10.3389/fnut.2022.1036655

**Published:** 2022-11-10

**Authors:** Jiyoung Bae, Yang Yang, Xinyun Xu, Jamie Flaherty, Haley Overby, Kelsey Hildreth, Jiangang Chen, Shu Wang, Ling Zhao

**Affiliations:** ^1^Department of Nutrition, The University of Tennessee, Knoxville, Knoxville, TN, United States; ^2^Department of Public Health, The University of Tennessee, Knoxville, Knoxville, TN, United States; ^3^College of Health Solutions, Arizona State University, Phoenix, AZ, United States

**Keywords:** naringenin, browning, brown adipogenesis, PPARγ, citrus flavanone

## Abstract

Identifying functional brown adipose tissue (BAT) has provided new hope for obesity treatment and prevention. Functional BAT includes classical BAT and brown-like adipose tissue converted from white adipose tissue. By promoting thermogenesis (i.e., heat production) *via* uncoupling protein 1 (UCP1), functional BAT can increase energy expenditure and aid obesity treatment and prevention. Naringenin (NAR) is a flavanone primarily found in citrus fruits. NAR has been reported to decrease body weight, increase energy expenditure in treated mice, and promote browning in human adipocytes. Here, we examined the effects of NAR on 3T3-L1 adipocytes’ browning and β-adrenergic agonist isoproterenol (ISO)-stimulated thermogenic activation and classical murine brown adipogenesis. In addition, we demonstrated the signaling pathways and involvement of peroxisome proliferator-activated receptor gamma (PPARγ) in the process. We found that NAR did not increase *Ucp1* mRNA expression at the basal (i.e., non-ISO stimulated) condition. Instead, it enhanced *Ucp1* and *Pgc-1*α up-regulation and thermogenesis under ISO-stimulated conditions in 3T3-L1 adipocytes. NAR promoted protein kinase A (PKA) activation and phosphorylation of p38 MAPK downstream of ISO stimulation and activated PPARγ. Pharmacological inhibition of either PKA or p38 and PPARγ knockdown attenuated *Ucp1* up-regulation by NAR. Moreover, NAR promoted brown adipogenesis by increasing lipid accumulation, brown marker expression, and thermogenesis in murine brown adipocytes, which was also attenuated by PPARγ knockdown. Together, our results suggest that NAR may promote the development of functional BAT in part through PPARγ activation. NAR’s role in combating human obesity warrants further investigation.

## Introduction

Obesity has become a pandemic across the globe over the past decades. In addition to well-recognized higher risks of developing many chronic diseases, such as diabetes, cardiovascular diseases, and some types of cancer (1), obesity is also associated with increased severity and mortality of coronavirus disease (COVID-19), an ongoing pandemic infectious disease caused by SARS-CoV-2 virus (2, 3).

White adipose tissue (WAT) and brown adipose tissue (BAT) contribute to energy homeostasis. BAT is responsible for non-shivering thermogenesis *via* uncoupling protein 1 (UCP1), leading to energy expenditure. In addition, animal and *in vitro* studies have demonstrated inducible brown-like adipocytes, also known as beige adipocytes, in WAT. These brown-like adipocytes can be generated by β-adrenergic stimulation from cold exposure or synthetic β-adrenergic receptor (β-AR) agonists (4–6). Activation of peroxisome proliferator-activated receptor gamma (PPARγ) by its agonists, such as rosiglitazone (ROSI), also promotes browning and enhances thermogenic activation induced by β-adrenergic stimulation (7–10). It is well recognized that functional BAT, including classical brown and brown-like adipose tissue, exists in humans (11–14). Their mass or activities negatively correlate with body mass index, total, or visceral fat mass (15), blood glucose, and HbA1c levels (16). Moreover, cold exposure or daily intake of capsinoids increases BAT activation and energy expenditure and decreases body fat mass in human subjects (17). Therefore, strategies that promote functional BAT are promising for combating human obesity.

Naringenin (4′,5,7-trihydroxyflavanone, NAR), a flavanone commonly found in citrus fruits, has been reported with many beneficial health effects, including anti-inflammatory, anti-oxidative, and anti-carcinogenic effects (18–22). In addition, NAR was reported to activate PPARγ and other nuclear receptors (23). PPARγ activation by its agonist enhances both browning and brown adipogenesis (24, 25). Therefore, it is conceivable that NAR may induce browning and brown adipogenesis to confer anti-obesity benefits. Indeed, NAR decreased body weight with increases in energy expenditures in both chow-fed lean and high-fat diet-fed obese Ldlr-/-mice (26, 27) and reversed the attenuation of *Ucp1* mRNA expression in the BAT by a high-fat diet in rats (28). Consistently, NAR induced thermogenic *UCP1*, *PGC1α*, and *PGC1*β expression in human white adipocytes (29). However, the direct effects of NAR on thermogenic activation by a β-adrenergic agonist in brown-like adipocytes and classical brown adipogenesis remain unknown, and the underlying molecular mechanisms are not completely understood.

In this report, we examined the effects of NAR on browning and thermogenic activation by isoproterenol, a β-adrenergic receptor agonist, in 3T3-L1 white adipocytes and classical brown adipogenesis in murine brown pre-adipocytes. We further explored the molecular pathways by which NAR promotes thermogenic activation and the role of PPARγ in the process.

## Materials and methods

### Reagents

Naringenin, ROSI, insulin, 3-isobutyl-L-methylxanthine, dexamethasone, and isoproterenol (ISO) were from MilliporeSigma (St. Louis, MO, USA). Calf serum (CS) was purchased from Hyclone (Logan, UT, USA), and fetal bovine serum (FBS) was purchased from Bio-techne (Minneapolis, MN, USA). The pharmacological inhibitors for p38 (SB203580) and PKA (H-89) were from Tocris Bioscience (Ellisville, MI, USA). Anti-phospho-p38 (Thr180/Tyr182) (Catalog# 9211, RRID:AB_331641), anti-p38 (Catalog# 9212, RRID:AB_330713), and anti-ERK1/2 (Catalog# 4695, RRID:AB_390779) antibodies and horseradish peroxidase-conjugated goat anti-rabbit were from Cell Signaling Technology (Danvers, MA, USA). Anti-UCP1 (Catalog# U6382, RRID:AB_261838) was purchased from Sigma Aldrich (St. Louis, MO, USA); Anti-PGC1α antibody (Catalog# AB3242, RRID:AB_2268462) was purchased from Millipore (Temecula, CA, USA). Other reagents, if not specified, were purchased from MilliporeSigma.

Naringenin was dissolved in dimethyl sulfoxide (DMSO) to make 50 mM stock, followed by dilution in DMSO to make 0, 5, 10, and 20 mM stocks for treatment. The final DMSO concentration in the cell culture medium was 0.1% (v/v).

### Cell culture and treatment

Murine 3T3-L1 cells were grown in DMEM containing 10% calf serum at 37°C humidified incubator with 5% CO_2_. The cells were differentiated as described (30). Briefly, at the confluence (day 0), the cells were induced to differentiate in DMEM containing 10% FBS, 0.5 mM/L 3-isobutyl-1-methylxanthine, 1 μM/L dexamethasone, and 10 μg/mL insulin for 3 days, followed by DMEM containing 10% FBS and 10 μg/mL of insulin for 2 days. The cells were then kept in DMEM containing 10% FBS until day 7, when the cells were fully differentiated.

To study NAR’s effects, 3T3-L1 cells were differentiated in the presence or absence of various doses of NAR from day 0. Fresh NAR was replaced at each change of the media. ROSI (1 μM) served as a positive control. On day 7, the cells were treated with isoproterenol (ISO, 1 μM) or the vehicle control (H_2_O) for 6 h for mRNA analysis or 24 h for protein analysis. In a separate experiment, the cells were pre-treated with the pharmacological inhibitor of PKA (H-89), p38 (SB203580), or the vehicle control (DMSO) for 1 h before ISO treatment, as indicated in the figure legends.

Murine primary stromal cells were isolated from the white fat pads (both the subcutaneous and epididymal fat) of C57BL/6 J male mice (12 weeks old) and differentiated as described with modification (31). The animal study has been approved by the University of Tennessee Knoxville Institutional Animal Care and Use Committee under animal protocol 2,320. After reaching confluence (day 0), the primary stromal cells were differentiated in DMEM containing 10% FBS, 1 μM/L dexamethasone, 0.5 mM/L 3-isobutyl-1-methylxanthine and 10 μg/mL insulin for 7 days, followed by DMEM containing 10% FBS for another 7 days. NAR (10 μM) or the vehicle control (DMSO) was added to the media from day 0. The treatments were replaced at each change of media.

Murine brown pre-adipocyte cell line was a gift from Dr. Klein (32). Murine brown pre-adipocytes were grown in DMEM containing 20% FBS until they reached confluence (day 0). The cells were differentiated in DMEM containing 20% FBS, 1 nM T3, and 20 nM insulin (differentiation media) for 6 days, with media change every 2 days. To study NAR’s effects, NAR (10 μM), the vehicle control (DMSO), or ROSI (1 μM, a positive control) were added to the media from day 0. The treatments were replaced at each change of media.

### Peroxisome proliferator-activated receptor gamma knockdown (PPARγ-KD)

3T3-L1 with PPARγ-KD or a scrambled non-targeting control have been described elsewhere (33). Murine brown pre-adipocytes with PPARγ-KD or a scrambled non-targeting control were generated by lentiviral shRNA infection. Briefly, murine brown pre-adipocytes were plated at ∼50% confluence in a 6-well plate overnight. The cells were then infected with MISSION lentiviral shRNA transduction-ready particles against mouse *Pparg* or a scrambled non-targeting control according to the manufacturer’s instructions (MilliporeSigma). Stably infected cells were selected by puromycin for 2 weeks.

### Protein kinase A activity

On day 7, 3T3-L1 adipocytes treated with NAR (10 μM), the vehicle control, or ROSI (1 μM) were serum starved with 0.25% FBS containing DMEM for 1 h. Then the cells were treated with ISO (1 μM) or the vehicle control (H_2_O) for an additional 6 h. Protein kinase A (PKA) activities were measured from total cell lysates using the DetectX PKA activity kit (Arbor Assays, Ann Arbor, MI, USA) according to the manufacturer’s instructions.

### Western blot analysis

**FIGURE 1:**
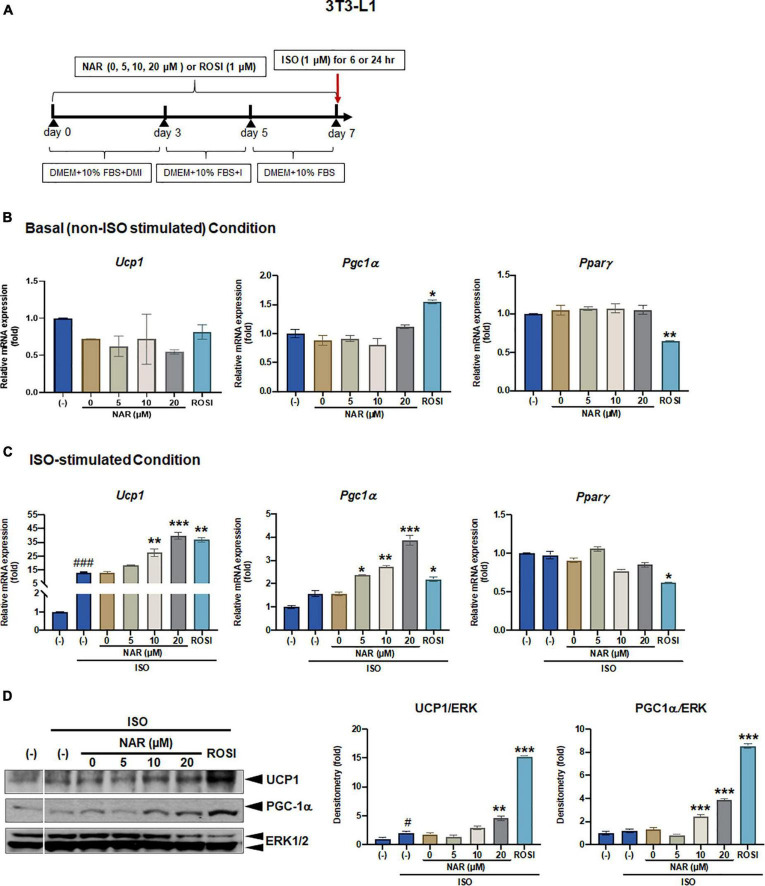
Naringenin dose-dependently enhances UCP-1 expression in isoproterenol (ISO)-stimulated 3T3-L1 adipocytes. 3T3-L1 cells were differentiated in the presence or absence of NAR (5, 10, 20 μM) for 7 days. ROSI (1 μM) was included as a positive control. On day 7, the cells were stimulated with isoproterenol (ISO, 1 μM) or the vehicle control (H_2_O) for 6 h for mRNA analysis or 24 h for protein analysis. **(A)** A diagram of the cell treatment and timeline. As described in the Materials and Methods, DMEM + 10% FBS + DMI refer to DMEM containing 10% FBS, 0.5 mM/L 3-isobutyl-1-methylxanthine, 1 μM/L dexamethasone, and 10 μg/mL insulin. DEME + 10% FBS + I refer to DMEM containing 10% FBS and 10 μg/mL insulin. DMEM + 10% FBS refer to DMEM containing 10% FBS. **(B,C)** Relative mRNA levels of *Ucp1, Pgc1*α, and *Ppar*γ at the basal **(B)** and ISO-stimulated conditions **(C)**. mRNA expression is presented relatively to the loading control *36b4*. **(D)** Protein expression of UCP1, PGC1α, and the loading control ERK1/2. Bar graphs show the densitometry of each protein to the loading control ERK1/2. Data = Mean ± SEM (*n* = 3). *, **, ***p < 0.05, *p* < 0.01, and *p* < 0.001 compared to the 0 μM NAR (i.e., DMSO) samples, respectively **(B–D)**. #, ###*p* < 0.05, *p* < 0.001 compared to the non-ISO stimulated (–) samples, respectively **(C,D)**.

Total cell lysates were prepared with lysis buffer (Cell Signaling, Danvers, MA, USA). Protein concentrations were measured by a BCA assay kit (Thermo Fisher Scientific, Waltham, MA, USA). Total cell lysates were subjected to 10% SDS-PAGE and transferred overnight to polyvinylidene difluoride membranes (Bio-Rad, Hercules, CA, USA). The membranes were blocked in 137 mM NaCl, 20 mM Tris⋅HCl, and 0.1% Tween 20 (pH 7.4) solution with 5% non-fat milk, followed by immunoblotting with primary antibodies at 4°C overnight and secondary antibody conjugated with horseradish peroxidase for 1 h. ERK1/2 served as the loading control for Figures 1, 6, 7, as previously reported (32–34). The signals were produced by incubating the membranes with ECL Western blot detection reagents (GE Healthcare, Piscataway, NJ, USA) and captured by X-ray films or ChemiDoc Imaging Systems (Bio-Rad, Hercules, CA, USA). The membranes were stripped in the stripping buffer (100 mM 2-mercaptoethanol, 62.5 mM Tris-HCl, and 2% SDS) for 10 min at 50°C and re-probed with different antibodies.

### Total RNA isolation and semi-quantitative reverse-transcription PCR analysis

Total RNA was isolated with TRI reagent (Molecular Research Center, Cincinnati, OH, USA) according to the manufacturer’s instructions. Total RNA abundance was measured by a NanoDrop ND-1,000 spectrophotometer (NanoDrop Technologies, Wilmington, DE, USA). Reverse transcription was performed using a High Capacity cDNA Reverse Transcription kit (Thermo Scientific, Waltham, MA, USA) according to the manufacturer’s instructions. Analysis of mRNA expression of the target genes and the housekeeping gene *36b4* [encodes acidic ribosomal phosphoprotein P0 (RPLP0)] were carried out using Absolute Blue QPCR SYBR Green ROX mix (Thermo Fisher Scientific, Waltham, MA, USA). PCR reactions were carried out in 96-well plates in an ABI 7300HT instrument. The conditions were set at 50°C 2 min and 95°C 15 min, followed by 40 cycles of 95°C 15 s/60°C 1 min. Relative gene expression was calculated using the 2^–ΔΔCt^ method (35), which normalizes against *36b4*. The primer sequences were reported in our previous study (25).

### Reporter gene assays

3T3-L1 cells were seeded at 2.5 × 10 (4) per well in 24-well plates overnight. The following day, the cells were transiently transfected with either PPRE X3-TK-Luc, a PPAR response element driven luciferase reporter, a gift from Dr. Bruce Spiegelman (Addgene plasmid # 1015^1^; RRID:Addgene_1015) (36) or murine PPARγ *trans*-activation reporters that include murine PPARγ ligand binding domain (LBD) linked to the Gal4 DNA binding domain (DBD) (mPPARγ-Gal4) and a reporter with an upstream activating sequence linked to luciferase, 4xUAS-TK-Luc (TK: thymidine kinase) (37) and β-galactosidase (β-gal) plasmid with Fugene HD transfection reagent (Promega, Madison, WI, USA). The cells were then treated with NAR, DMSO, or ROSI for 18 h. Luciferase and β-gal activities were measured from the cell lysates with GloMax Multi Detection System (Promega, Madison, WI, USA). Relative luciferase activities were presented by normalizing the luciferase activities with β-gal activities.

### Cellular bioenergetics measurements

3T3-L1 cells were differentiated in the presence or absence of NAR (10 μM) or ROSI (1 μM) for 6 days before they were seeded at 20,000 cells per well into XFe24 assay plates in DMEM containing 10% FBS overnight. Murine brown pre-adipocytes were differentiated in the presence or absence of NAR (10 μM) or ROSI (1 μM) for 4 days before they were seeded at 20,000 cells per well into XFe24 assay plates in the differentiation media overnight.

To start cellular bioenergetics measurements, cells were washed three times with XF assay buffer (DMEM without NaHCO_3_, 10 mM glucose, 2 mM pyruvate, and 2 mM GlutaMAX, and 2% bovine serum albumin, pH 7.4). The cells were then equilibrated at 37°C in a non-CO_2_ incubator for 1 h in the XF assay buffer. Oxygen consumption rates (OCR) were measured in an XFe24 Extracellular Flux Analyzer (Agilent, Santa Clara, CA, USA). To carry out mitochondria stress tests, ISO (10 μM, only for 3T3-L1 adipocytes), oligomycin (1 μM), carbonyl cyanide-ptrifluoromethoxyphenylhydrazone (FCCP; 6.5 μM), rotenone/antimycin A (1 μM each) were injected in sequential order, and three readings were taken after each injection. OCR readings were recorded by XFe24 software. OCR linked to proton leak and ATP production, coupling efficiency, and maximal respiration were calculated according to the manufacturer’s instructions.

### Oil red O staining

Lipid accumulation in the differentiated brown adipocytes was assessed by oil red O (ORO) staining and ORO absorbance, as described (25).

### Statistical analysis

All data are shown as mean ± SE. Triplicates were performed in each experiment. Statistical analysis was conducted using Prism 9.3.0 (GraphPad Software, San Diego, CA, USA). One-way ANOVA with repeated measures followed by multiple comparisons test (Student-Newman-Keuls method) was used to detect significant differences in group mean between the treatment groups or between time points. Two-way ANOVA was used to detect differences between treatment groups. Student’s *t*-tests were used as needed. The level of significance was set at *p* < 0.05.

## Results

### Naringenin dose-dependently enhances uncoupling protein 1 expression in isoproterenol-stimulated white adipocytes

To examine the effects of NAR on browning, 3T3-L1 cells were differentiated in the presence of increasing concentrations of NAR (5, 10, 20 μM) or its vehicle control DMSO. ROSI served as a positive control (Figure 1A). At basal (i.e., non-stimulated) condition (Figure 1B), ROSI induced mRNA expression of established brown specific markers *Ucp1*, *Pgc1*α, and suppressed *Pparγ* mRNA expression, as reported (9). In contrast, NAR did not change the mRNA expression of *Ucp1, Pgc1*α, and *Pparγ* when used up to 20 μM at the basal condition (Figure 1B). When stimulated with ISO, NAR dose-dependently enhanced ISO-induced *Ucp1* mRNA expression (Figure 1C). Similar effects were seen in *Pgc1α*, but not *Pparγ* (Figure 1C). ROSI also significantly enhanced ISO-induced *Ucp1* and *Pgc1α* up-regulation but suppressed *Pparγ* mRNA expression (Figure 1C). Consistently, NAR dose-dependently increased UCP1 and PGC1α protein expression in ISO-stimulated 3T3-L1 adipocytes (Figure 1D and Supplementary Figure 1A).

**FIGURE 2:**
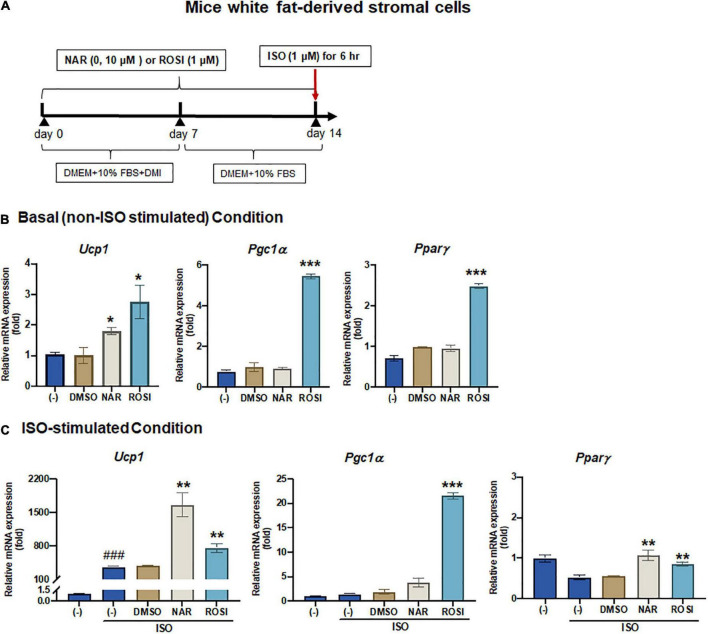
Naringenin enhances basal and isoproterenol (ISO)-stimulated up-regulation of *Ucp1* mRNA expression in primary white adipocytes differentiated from mice white fat-derived stromal cells. Mice white fat-derived primary stromal cells were induced to differentiate in the presence or absence of NAR (10 μM) or ROSI (1 μM) for 14 days. On day 14, the cells were stimulated with ISO (1 μM) or the vehicle control for 6 h for mRNA analysis. **(A)** A diagram of the cell treatment and timeline. As described in the Materials and Methods, DMEM + 10% FBS + DMI refer to DMEM containing 10% FBS, 0.5 mM/L 3-isobutyl-1-methylxanthine, 1 μM/L dexamethasone, and 10 μg/mL insulin. DMEM + 10% FBS refer to DMEM containing 10% FBS. **(B,C)** Relative mRNA levels of *Ucp1, Pgc1*α, and *Ppar*γ at the basal **(B)** and ISO-stimulated conditions **(C)**. mRNA expression is presented relatively to the loading control *36b4.* Data = Mean ± SEM (*n* = 3). *, **, ***p < 0.05, *p* < 0.01, and *p* < 0.001 compared to the DMSO samples, respectively **(B,C)**. ###*p* < 0.001 compared to the non-ISO stimulated (–) samples **(C)**.

Since it has been reported that the physiologically achievable serum level of NAR in human subjects is ∼8 μM (38), we, therefore, focused our studies on NAR at 10 μM. We further examined the effects of NAR on murine primary stromal cells derived from white fat pads from C57BL/6 J mice. Primary stromal cells derived from the mice white fat pads were differentiated in the presence or absence of NAR (10 μM) or ROSI for 14 days (Figure 2A). NAR significantly increased *Ucp1* mRNA expression at the basal condition (Figure 2B) and enhanced ISO-stimulated up-regulation of *Ucp1* and *Pparγ* mRNA in these primary adipocytes (Figure 2C).

### Naringenin enhances protein kinase A activation and phosphorylation of p38 MAPK in isoproterenol-stimulated 3T3-L1 adipocytes

**FIGURE 3:**
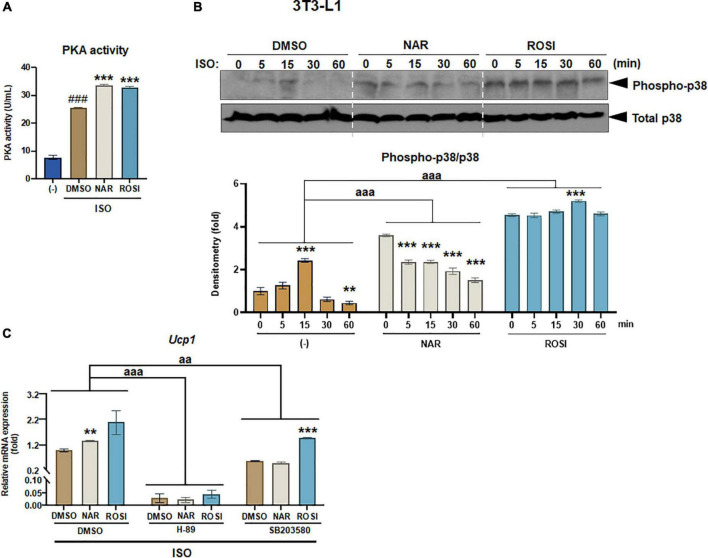
Naringenin enhances protein kinase A (PKA) activation and phosphorylation of p38 MAPK required for *Ucp1* up-regulation in isoproterenol (ISO)-stimulated 3T3-L1 adipocytes. 3T3-L1 cells were differentiated in the presence or absence of NAR (10 μM) or ROSI (1 μM) for 7 days. On day 7, the cells were stimulated with ISO (1 μM) or the vehicle control for 6 h for PKA activity analysis (A) or 1 h for analysis of phosphorylation of p38 (B). In a separate experiment, the cells were pretreated with the pharmacological inhibitors of PKA (H-89) and p38 (SB203580) or the vehicle control (DMSO) for 1 h before ISO stimulation for 6 h for mRNA expression analysis (C). **(A)** PKA activity. **(B)** p38 phosphorylation analysis. **(C)** Effects of PKA and p38 inhibitors on ISO-stimulated *Ucp1* up-regulation by NAR. *Ucp1* mRNA expression is presented relatively to the loading control *36b4.* Data = Mean ± SEM (*n* = 3). ###*p* < 0.001 compared to the non-ISO stimulated (–) samples **(A)**. *, **, ***p < 0.05, *p* < 0.01, and *p* < 0.001 compared to the ISO-stimulated time 0 **(B)** or DMSO samples **(C)** within each group, respectively. aa and aaa, *p* < 0.01 and *p* < 0.001 compared to the DMSO group, respectively, by two-way ANOVA **(B,C)**.

β-adrenergic activation induced by cold exposure increases cAMP levels, leading to activation of PKA and downstream p38 MAPK phosphorylation and, consequently, UCP1 protein expression in brown adipocytes (39). To understand the molecular mechanisms by which NAR increases ISO-stimulated UCP1 expression in 3T3-L1 adipocytes, we explored the effects of NAR on PKA activation and p38 phosphorylation in ISO-stimulated 3T3-L1 adipocytes (Figure 3). ISO stimulation led to ∼3-fold increase of PKA activities, compared with the non-stimulated controls (Figure 3A). Both NAR and ROSI significantly enhanced PKA activation induced by ISO (Figure 3A).

We further examined p38 phosphorylation downstream of PKA activation in ISO-stimulated 3T3-L1 adipocytes (Figures 3B,C). As shown, ISO induced a peak of p38 phosphorylation at 15 min upon the stimulation in the control (DMSO treated) cells (Figure 3B). In contrast, p38 phosphorylation was significantly higher at time 0 but gradually decreased upon the stimulation in the NAR treated cells. ROSI induced the highest p38 phosphorylation at time 0 and a small peak at 30 min upon the stimulation in 3T3-L1 adipocytes (Figure 3B).

To examine whether PKA/p38 pathways underlie ISO-stimulated *Ucp1* up-regulation by NAR, we employed PKA and p38 pharmacological inhibitors. The PKA inhibitor H-89 blocked *Ucp1* mRNA expression at the basal condition and the up-regulation induced by NAR and ROSI (Figure 3C). The p38 inhibitor SB203580 also significantly attenuated the basal as well as NAR- and ROSI-induced, *Ucp1* mRNA expression, although to a less extent compared to H-89 (Figure 3C).

### Naringenin enhances *Ucp1* mRNA expression in isoproterenol-stimulated 3T3-L1 adipocytes through peroxisome proliferator-activated receptor gamma activation

**FIGURE 4:**
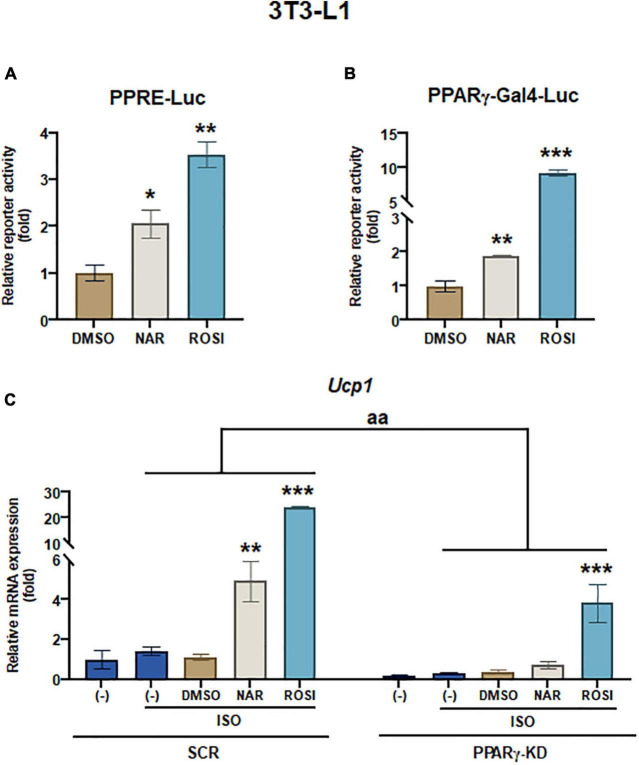
Naringenin activates PPRE and peroxisome proliferator-activated receptor gamma (PPARγ) reporters, and peroxisome proliferator-activated receptor gamma (PPARγ) knockdown attenuates the up-regulation of *Ucp1* mRNA expression by naringenin in isoproterenol (ISO)-stimulated 3T3-L1 adipocytes. **(A,B)** Effects of NAR on PPAR activation. 3T3-L1 cells were transiently transfected with PPRE-Luc **(A)** or murine PPARγ *trans*-activation reporter and the β-gal control plasmid **(B)** as described. Then the cells were treated with NAR, ROSI, or DMSO for 18 h, and the reporter activities were measured. Relative luciferase activities were calculated and presented as a fold with the DMSO sample set as 1. **(C)** Effects of PPARγ knockdown on ISO-stimulated *Ucp1* up-regulation by NAR. 3T3-L1 cells with PPARγ knockdown (PPARγ-KD) and a scrambled non-targeting control (SCR) were differentiated in the presence or absence of NAR (10 μM) or ROSI (1 μM) for 7 days. On day 7, the cells were stimulated with ISO (1 μM) for 6 h for mRNA analysis. *Ucp1* mRNA expression is presented relatively to the loading control *36b4*. Data = Mean ± SEM (*n* = 3). *, **, ***p < 0.05, *p* < 0.01, and *p* < 0.001 compared to the DMSO samples **(A,B)** or ISO-stimulated DMSO samples **(C)**, respectively. aa, *p* < 0.01 compared to the SCR control group by two-way ANOVA **(C)**.

It has been reported that as one of the downstream targets of the PKA/p38 pathway, PPARγ interacts with PGC1α and activates the PPRE site in the *Ucp1* promotor, leading to *Ucp1* transcription (39, 40). To understand whether NAR’s effects were specifically mediated through PPARγ in 3T3-L1 pre-adipocytes, we first performed reporter gene assays. At 10 μM, NAR activated PPRE-Luc reporter by ∼2-fold, whereas ROSI activated the reporter by ∼3.5-fold compared to the controls (Figure 4A). Moreover, NAR at the same concentration *trans*-activated PPARγ *via* its ligand binding domain in 3T3-L1 cells (Figure 4B), consistent with the previous report (23). Furthermore, using 3T3-L1 with PPARγ knockdown (PPARγ-KD) and the scrambled non-targeting control (SCR) we have generated (25), we found that ISO-induced *Ucp1* mRNA expression was significantly enhanced by NAR or ROSI in the SCR cells but was significantly attenuated in PPARγ-KD cells (Figure 4C).

### Naringenin enhances isoproterenol-stimulated mitochondrial respiration and uncoupling in 3T3-L1 adipocytes

**FIGURE 5:**
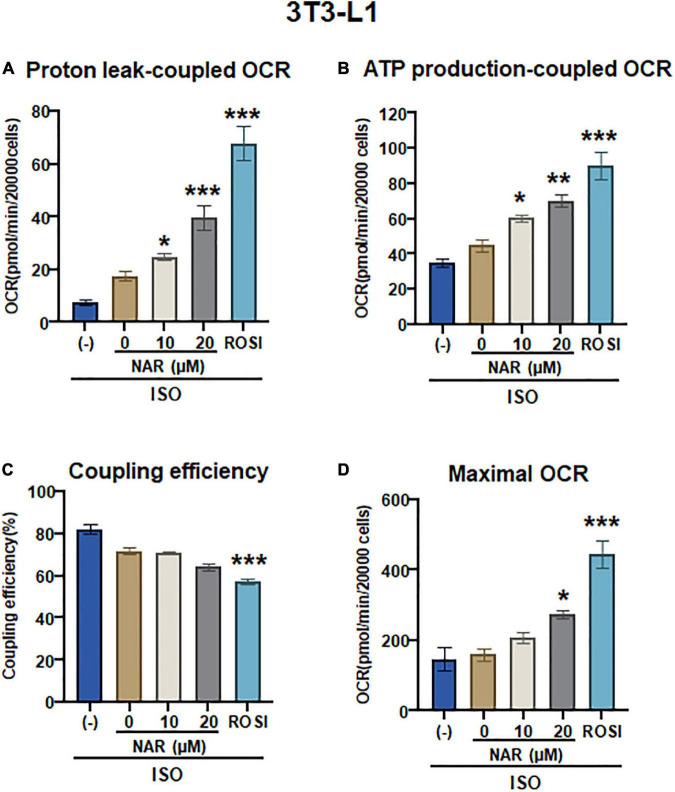
Naringenin enhances mitochondrial respiration and uncoupling in isoproterenol (ISO)-stimulated 3T3-L1 adipocytes. **(A–D)** 3T3-L1 cells were differentiated in the presence or absence of NAR (10 and 20 μM) or ROSI (1 μM) for 6 days. Then the cells were reseeded at 20,000 per well into an XFe24 assay plate. After 24 h, the cells were subjected to real-time OCR measurements using an XFe24 Extracellular Flux Analyzer. ISO was injected after three basal OCRs were taken, followed by oligomycin, FCCP, and rotenone/antimycin A injections as described. OCR linked to proton leak **(A)** and ATP production **(B)**, coupling efficiency **(C)**, and maximal respiration **(D)** were calculated and presented. Data = Mean ± SEM (*n* = 3–7). *, **, ***p < 0.05, *p* < 0.01, and *p* < 0.001 compared to the 0 μM NAR sample, respectively.

To determine whether UCP1 up-regulation by NAR upon ISO stimulation leads to increases in mitochondrial respiration and uncoupling, we measured oxygen consumption rates (OCR) in mitochondrial stress tests in 3T3-L1 adipocytes that were differentiated in the presence of NAR, the vehicle control, or ROSI using an XFe24 Extracellular Flux Analyzer (Figure 5 and Supplementary Figure 1B). We found that NAR dose-dependently enhanced OCR linked to proton leak (i.e., uncoupling) (Figure 5A) and ATP production (Figure 5B) and increased maximal OCR (Figure 5D) while had no significant effects on coupling efficiency (Figure 5C) in ISO-stimulated 3T3-L1 adipocytes. Note that significant increases in OCR linked to proton leak and ATP production by NAR were detected starting at 10 μM.

**FIGURE 6:**
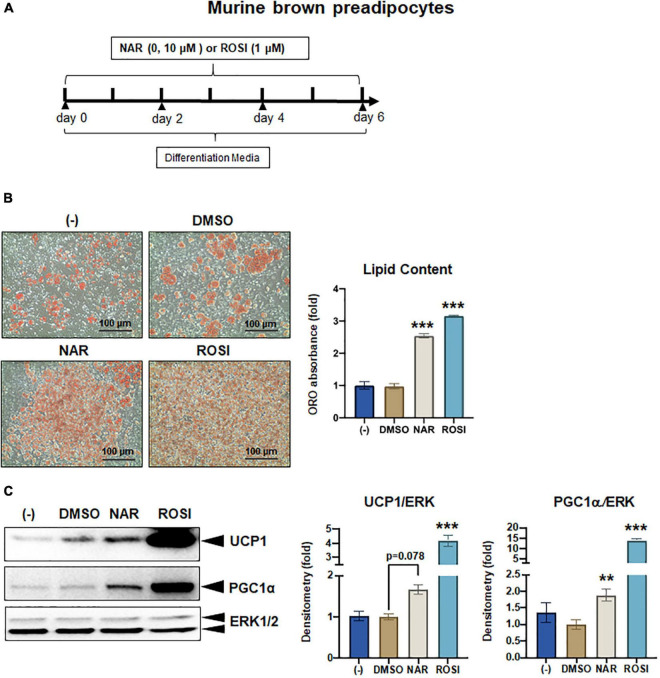
Naringenin promotes brown adipogenesis and thermogenic protein expression in murine brown adipocytes. Murine brown pre-adipocytes were differentiated in the presence or absence of NAR (10 μM) or ROSI (1 μM) for 6 days. **(A)** A diagram of the cell treatment and timeline. **(B)** Oil red O (ORO)-stained cell morphology and ORO absorbance. Scale bar = 100 μm. **(C)** Protein expression of thermogenic genes UCP1, PGC1α, and the loading control ERK1/2. Bar graphs show the densitometry of each protein normalized to the loading control ERK1/2. Data = Mean ± SEM (*n* = 3). *, **, ***p < 0.05, *p* < 0.01, and *p* < 0.001 compared to the DMSO samples, respectively.

**FIGURE 7:**
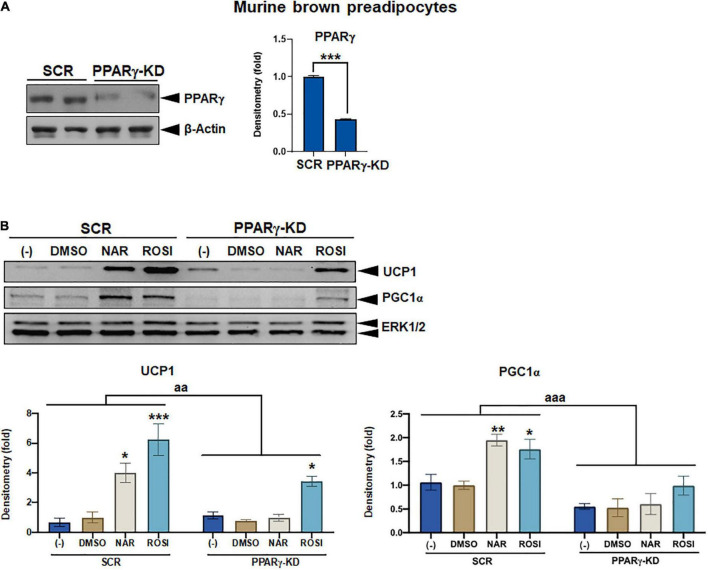
Attenuation of thermogenic protein expression by peroxisome proliferator-activated receptor gamma (PPARγ) knockdown in naringenin-treated murine brown adipocytes. **(A)** Knockdown efficiency in murine brown pre-adipocytes with PPARγ knockdown (PPARγ-KD) compared to the cells with a scrambled non-targeting control (SCR). Two individual clones were analyzed. Average knockdown efficiency is shown. **(B)** PPARγ-KD and SCR cells were differentiated in the presence or absence of NAR (10 μM) or ROSI (1 μM) for 6 days. Protein expression of thermogenic markers UCP1, PGC1α, and the loading control ERK1/2 are shown. Bar graphs show the densitometry of each protein normalized to the loading control ERK1/2. Data = Mean ± SEM (*n* = 3). *, **, ***p < 0.05, *p* < 0.01, and *p* < 0.001 compared to the SCR clones **(A)** or the DMSO samples within SCR or PPARγ-KD group **(B)**, respectively. aa and aaa, *p* < 0.01 and *p* < 0.001 compared to the SCR group, respectively, by two-way ANOVA **(B)**.

### Naringenin promotes brown adipogenesis and thermogenic protein expression in murine brown adipocytes through peroxisome proliferator-activated receptor gamma

As a part of functional brown adipose tissue, classical brown adipocytes are responsible for non-shivering thermogenesis in response to cold, leading to energy expenditure. However, despite the effects on browning, the effects of NAR on brown adipogenesis have not been reported. Here, we examined the effects of NAR on brown adipogenesis in a murine brown pre-adipocyte cell line. Murine brown pre-adipocytes were differentiated in the presence or absence of NAR or ROSI (Figure 6A). NAR at 10 μM significantly enhanced brown adipogenesis as indicated by the oil red O-stained cell morphology (Figure 6B left) and lipid accumulation (Figure 6B right) and increased protein expression of brown markers UCP1 and PGC1α (Figure 6C) and other general differentiation markers PPARγ, fatty acid binding protein 4 (FABP4), and perilipin (PLIN) (Supplementary Figure 2A). We further assessed the role of PPARγ in the process using the brown pre-adipocytes with stable knockdown of PPARγ (PPARγ-KD) and a scrambled non-targeting control (SCR). PPARγ-KD reduced endogenous PPARγ protein expression by ∼60% (Figure 7A). While NAR at 10 μM significantly increased UCP1 and PGC1α protein expression in the SCR cells, it did not cause significant changes in the PPARγ-KD cells (Figure 7B). ROSI’s effects were also significantly attenuated in PPARγ-KD cells as expected (Figure 7B).

### Naringenin enhances mitochondrial respiration and uncoupling in murine brown adipocytes

**FIGURE 8:**
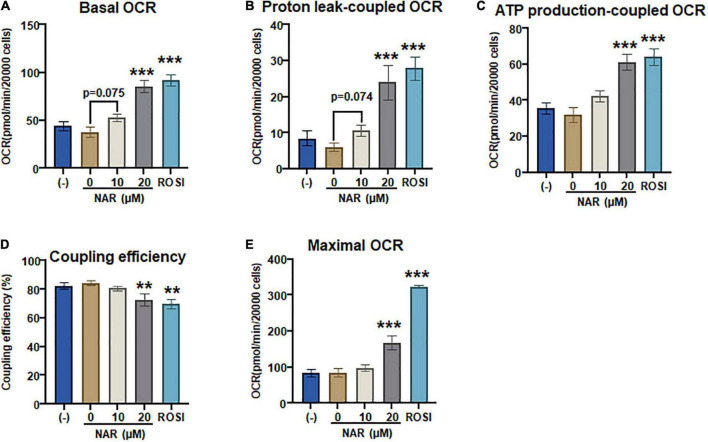
Naringenin enhances mitochondrial respiration and uncoupling in murine brown adipocytes. **(A–E)** Murine brown pre-adipocytes were differentiated in the presence or absence of NAR (10 and 20 μM) or ROSI (1 μM) for 4 days. Then cells were reseeded at 20,000 cells per well into an XFe24 assay plate. After 24 h, the cells were then subjected to real-time measurements of OCR coupled with a mitochondrial stress test using an XFe24 Extracellular Flux Analyzer as described. Basal OCRs **(A)**, OCR linked to proton leak **(B)** and ATP production **(C)**, coupling efficiency **(D)**, and maximal respiration **(E)** were calculated and presented. Data = Mean ± SEM (*n* = 3–7). *, **, ***p < 0.05, *p* < 0.01, and *p* < 0.001 compared to the 0 μM NAR samples, respectively.

To confirm that NAR also enhances mitochondrial respiration and thermogenesis in parallel with its effects on lipid accumulation and brown marker protein expression, we measured OCR coupled with mitochondrial stress tests in the murine brown adipocytes that were differentiated in the presence or absence of NAR or ROSI using the XFe24 Extracellular Flux Analyzer (Figure 8). We found that there were dose-dependent increases in the basal OCR (Figure 8A and Supplementary Figure 2B), OCR linked to proton leak (i.e., uncoupling) (Figure 8B) and ATP production (Figure 8C) and maximal OCR (Figure 8E) and a decrease in the coupling efficiency (Figure 8D) in brown adipocytes treated by NAR. However, significant changes in those measures were found by NAR at 20 μM only. As expected, ROSI significantly increased similar changes in those measures but to a greater extent compared to NAR at 20 μM (Figure 8).

## Discussion

Functional brown adipose tissue has become a novel target for obesity treatment and prevention. We report that NAR enhances ISO-stimulated UCP1 expression, mitochondrial respiration, and uncoupling in 3T3-L1 adipocytes. NAR enhances ISO-stimulated PKA activation and phosphorylation of p38, accompanied by PPARγ activation. Moreover, NAR enhances murine brown adipogenesis and increases brown adipocytes’ mitochondrial respiration and uncoupling. We further demonstrate that PPARγ is required for enhanced *Ucp1* expression in 3T3-L1 adipocytes and brown adipocytes by NAR.

3T3-L1 cells, a commonly used white fat cell model, have been used to identify and characterize strategies to induce browning (41–47). To our knowledge, this is the first report that NAR, a dietary bioactive compound, enhances ISO-stimulated thermogenic activation (*Ucp1* expression and mitochondrial uncoupling) in 3T3-L1 adipocytes at 10 μM, a level that is achievable through dietary consumption in human subjects (38).

The hallmark of brown-like adipocytes is inducible UCP1 expression and thermogenesis upon β adrenergic stimulation, such as cold exposure. As a β adrenergic receptor agonist, ISO has been used to induce thermogenic activation in brown (32, 48) and brown-like adipocytes (33, 41, 44, 48). It was reported that ISO induced increases in *Ucp1* mRNA expression in 3T3-L1 adipocytes (41). Therefore, our findings are consistent with the report and further highlight the browning capacity of NAR as a dietary factor in enhancing ISO-stimulated *Ucp1* expression in 3T3-L1 adipocytes. Moreover, we report that NAR at 10 μM, a reported dietary achievable dose in human subjects (29, 38), enhances *Ucp1* mRNA expression at both basal (non-ISO stimulated) and ISO-stimulated conditions in the primary white adipocytes differentiated from stromal cells isolated from mice white fat pads. These findings are consistent with the reported increase of UCP1 expression by NAR at the same dose in human white adipocytes under non-ISO-stimulated conditions (29). The effects of NAR on ISO-stimulated UCP1 up-regulation in human white adipocytes were not reported in that study and, therefore, warrant further investigation.

**FIGURE 9:**
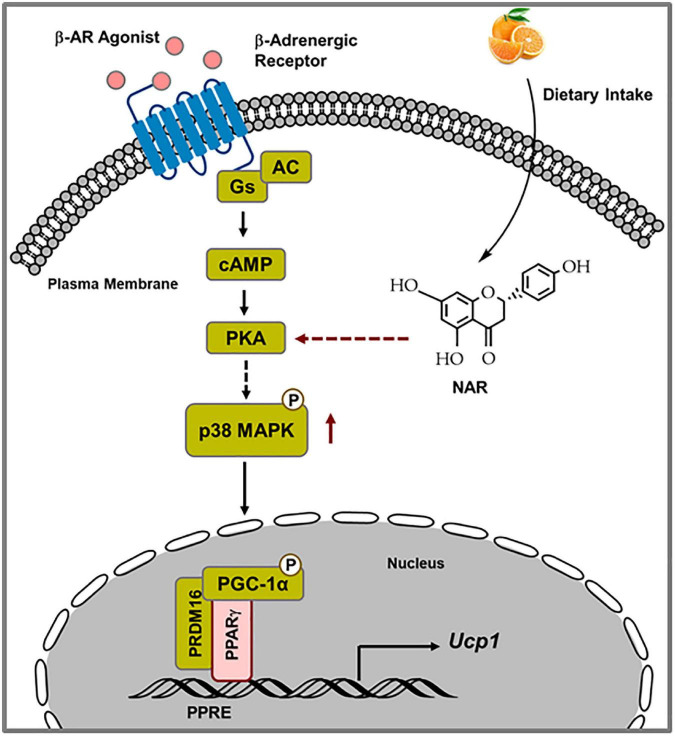
Schematic diagram illustrating the proposed mechanisms by which naringenin enhances isoproterenol (ISO)-stimulated UCP1 expression in 3T3-L1 adipocytes. Upon ISO binding to the β-adrenergic receptor (AR), cAMP is produced through Gs-coupled adenylyl cyclase (AC), leading to PKA activation. Through yet unknown steps, PKA activation results in p38 phosphorylation and subsequent phosphorylation and activation of downstream targets, such as PGC1α. Activated PGC1α co-activates PPARγ on the PPRE site in the promoter of the *Ucp1* gene, leading to enhanced *Ucp1* transcription. NAR enhances ISO-stimulated PKA activation and p38 phosphorylation and further activates PPARγ, leading to increased *Ucp1* up-regulation upon ISO stimulation in 3T3-L1 adipocytes.

The findings that NAR does not induce *Ucp1* mRNA expression at the basal conditions but enhances *Ucp1* expression and mitochondrial uncoupling in response to ISO in 3T3-L1 adipocytes prompted us to investigate the mechanisms by which NAR enhances ISO-stimulated *Ucp1* up-regulation in these adipocytes. Upon adrenergic stimulation by cold or other β-AR agonists, cyclic AMP (cAMP) is produced through activated Gs protein coupled-adenylyl cyclase (AC) associated with β-AR, leading to PKA activation and subsequent p38 phosphorylation and activation (49, 50). Activated p38 further phosphorylates and activates target proteins, such as PGC1α [a coactivator of PPARγ on the PPAR response elements (PPRE) site], leading to up-regulation of *Ucp1* transcription (39, 40; Figure 9). We report, for the first time, that NAR at a dietary achievable dose enhances ISO-stimulated PKA activation in 3T3-L1 adipocytes. Moreover, NAR-treated 3T3-L1 adipocytes have a higher basal p38 phosphorylation before ISO stimulation. NAR enhances PPARγ transactivation in 3T3-L1 cells. Furthermore, we demonstrate that NAR’s effects on ISO-stimulated *Ucp1* up-regulation are attenuated by the inhibition of PKA and p38 and by PPARγ knockdown. Our results suggest that NAR may act through PKA/p38/PPARγ pathway to enhance ISO-stimulated *Ucp1* up-regulation in 3T3-L1 adipocytes (Figure 9).

As a well-known browning agent, ROSI is shown to enhance PKA activation and basal p38 phosphorylation, leading to up-regulation of ISO-stimulated *Ucp1* transcription, mitochondrial respiration, and uncoupling in 3T3-L1 adipocytes in our studies, consistent with a previous report that demonstrated enhanced cAMP levels and ISO-stimulated oxygen consumption in ROSI-treated white adipocytes (10). Our results shed new light on the mechanisms by which ROSI promotes the browning of white adipocytes.

For the first time, we also report that, similar to ROSI, NAR enhances brown adipogenesis with increased brown marker gene expression and mitochondrial respiration and uncoupling. We further demonstrate that PPARγ is required for the increased UCP1 expression by NAR in the brown adipocytes with PPARγ knockdown. Together, our results suggest that NAR promotes the development of functional brown adipocytes *in vitro* through PPARγ activation. Our results may help explain the increased energy expenditure found in NAR-treated mice (26, 27). However, whether there were significant increases in functional BAT mass or activities in those treated mice is unclear in those studies. Future studies on how NAR supplementation increases energy expenditure *in vivo* are warranted.

In conclusion, our results demonstrate that NAR at a dietary achievable dose enhances ISO-stimulated UCP1 up-regulation and mitochondrial respiration and uncoupling in 3T3-L1 adipocytes, possibly through enhancement of PKA/p38/PPARγ pathways downstream of ISO. Moreover, NAR also enhances cellular brown adipogenesis through PPARγ activation. Combined with other published reports, our results suggest that NAR may be beneficial in promoting the development of functional BAT. Further studies of NAR in promoting thermogenesis and energy expenditure to combat human obesity through enhancing functional BAT are warranted.

## Data availability statement

The original contributions presented in the study are included in the article/Supplementary material, further inquiries can be directed to the corresponding author.

## Ethics statement

The animal study was reviewed and approved by University of Tennessee Knoxville IACUC animal protocol 2320.

## Author contributions

JB and YY performed the experiments, data analysis, and wrote the manuscript. XX, JF, HO, KH, and JC performed the experiments and data analysis. SW contributed to the study designs and provided funding support. LZ wrote the manuscript and provided funding support. All authors have read and approved the manuscript.

## Footnotes

1
http://n2t.net/addgene:1015

## Supplementary material

Supplementary Figure 1 The effects of Naringenin on UCP1 and PGC1α protein expression and mitochondrial respiration under ISO-stimulated conditions in 3T3-L1 adipocytes. 3T3-L1 cells were differentiated in the presence or absence of NAR (5, 10, 20 μM) for 7 days. ROSI (1 μM) was included as a positive control. **(A)** The cells were stimulated with isoproterenol (ISO, 10 μM) or the vehicle control (H_2_O) for 24 h. Protein samples were prepared, and UCP1, PGC1α, and ERK1/2 protein expression are shown. **(B)** The cells were reseeded at 20,000 per well into an XFe24 assay plate. After 24 h, the cells were subjected to real-time OCR measurements using an XFe24 Extracellular Flux Analyzer as described in Figure 5. OCR readings over time are shown.

Supplementary Figure 2 Naringenin increases protein expression of general differentiation markers and mitochondrial respiration in murine brown adipocytes. Murine brown pre-adipocytes were differentiated in the presence or absence of NAR (10 μM) or ROSI (1 μM). **(A)** After 6 days, protein expression of PPARγ, FABP4, perilipin (PLIN), and the loading control ERK1/2 are shown (left panel). Bar graphs show the densitometry of each gene normalized to the loading control ERK1/2 (right panels). Data = Mean ± SEM (*n* = 3). *, ^**^, ^***^*p* < 0.05, *p* < 0.01, and *p* < 0.001 compared to the DMSO samples, respectively. **(B)** The cells were reseeded at 20,000 cells per well on day 4 into an XFe24 assay plate. After 24 h, the cells were then subjected to OCR measurements using an XFe24 Extracellular Flux Analyzer as described in Figure 8. OCR readings over time are shown.
