# Supplementary figures and images for: Naringenin, a citrus flavanone, enhances browning and brown adipogenesis: Role of peroxisome proliferator-activated receptor gamma
Source: Front Nutr. 2022 Nov 10;9:1036655. doi: 10.3389/fnut.2022.1036655 (PMC9686290; doi:10.3389/fnut.2022.1036655)

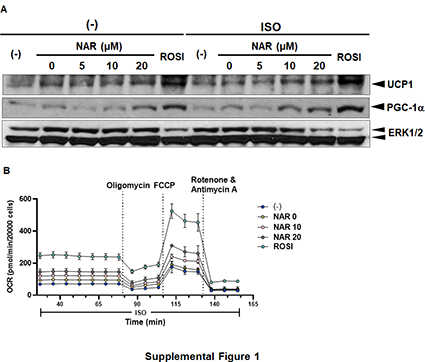

Supplement: Supplementary Figure 1 — The effects of Naringenin on UCP1 and PGC1α protein expression and mitochondrial respiration under ISO-stimulated conditions in 3T3-L1 adipocytes. 3T3-L1 cells were differentiated in the presence or absence of NAR (5, 10, 20 μM) for 7 days. ROSI (1 μM) was included as a positive control. (A) The cells were stimulated with isoproterenol (ISO, 10 μM) or the vehicle control (H2O) for 24 h. Protein samples were prepared, and UCP1, PGC1α, and ERK1/2 protein expression are shown. (B) The cells were reseeded at 20,000 per well into an XFe24 assay plate. After 24 h, the cells were subjected to real-time OCR measurements using an XFe24 Extracellular Flux Analyzer as described in Figure 5. OCR readings over time are shown. [file Image_1.TIF]

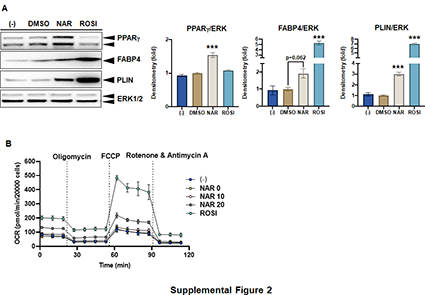

Supplement: Supplementary Figure 2 — Naringenin increases protein expression of general differentiation markers and mitochondrial respiration in murine brown adipocytes. Murine brown pre-adipocytes were differentiated in the presence or absence of NAR (10 μM) or ROSI (1 μM). (A) After 6 days, protein expression of PPARγ, FABP4, perilipin (PLIN), and the loading control ERK1/2 are shown (left panel). Bar graphs show the densitometry of each gene normalized to the loading control ERK1/2 (right panels). Data = Mean ± SEM (n = 3). *, **, ***p < 0.05, p < 0.01, and p < 0.001 compared to the DMSO samples, respectively. (B) The cells were reseeded at 20,000 cells per well on day 4 into an XFe24 assay plate. After 24 h, the cells were then subjected to OCR measurements using an XFe24 Extracellular Flux Analyzer as described in Figure 8. OCR readings over time are shown. [file Image_2.TIF]
